# *GALC* mutations in Chinese patients with late-onset Krabbe disease: a case report

**DOI:** 10.1186/s12883-019-1345-z

**Published:** 2019-06-11

**Authors:** Shunzhi Zhuang, Lingen Kong, Caiming Li, Likun Chen, Tingting Zhang

**Affiliations:** Department of Neurology, the First People’s Hospital of Huizhou city, 20# Sanxin south Road, Huizhou, 516003 Guangdong Province China

**Keywords:** Krabbe disease, Late-onset, Galactocerebrosidase, *GALC* gene, Brain MRI

## Abstract

**Background:**

Krabbe disease (also known as globoid cell leukodystrophy) cause by a deficiency of the enzyme β-galactocerebrosidase (galactosylceramidase, GALC). The deficiency of GALC leads to accumulation of galactosylceramide and psychosine, the latter GALC substrate having a potential role in triggering demyelination. Typically, the disease has an infantile onset, with rapid deterioration in the first few months, leading to death before the age of 2 years. The late onset forms (late-infantile, juvenile, and adult forms) are rare with variable clinical outcomes, presenting spastic paraplegia as the main symptom.

**Case presentation:**

We recruited a family with two affected individuals. The proband (Patient 1), a 25-year-old male, was presented with slow progressive symptoms, including spastic gait disturbance and vision loss since the 5th year of life. His elder sister (Patient 2), became wheelchair-bound and demented at the age of 22 years. Brain magnetic resonance imaging (MRI) showed increased signal intensity in the white matter along with the involvement of the bilateral corticospinal tracts. GALC deficiency was confirmed by biochemical analysis. DNA sequencing revealed two mutations (c.865G > C: p. G289R and c.136G > T: p. D46Y) in *GALC*. The clinical characteristics, brain MRI, biochemical and molecular findings led to the diagnosis of Krabbe disease.

**Conclusion:**

Clinical and neuroimaged signs, positive enzymatic analysis and molecular data converged to definite diagnosis in this neurodegenerative disease.

## Background

Krabbe disease (MIM 245200) is a rare inherited metabolic, neurodegenerative disease, due to the deficiency of the enzyme GALC. It is a lysosomal hydrolase, and its deficiency leads to accumulation of galactosylceramide and psychosine. The latter of these GALC substrates is cytotoxic at enhanced concentrations which seem to explain rapid degeneration of myelin-generating cells in Krabbe disease. This severe lysosomal storage disorder starts in the classical early-infantile form with a rapid downhill course around the first 6 months of life [1]. Compared to this acute disease form, the late-onset forms have a slower clinical progression [2]. Depending on the time of disease onset, the late-onset forms of Krabbe disease are categorized into [3]: late-infantile (6 months to 3 years), juvenile (3-8 years), and adult [4]. Cerebral MRI, especially on T2 W scans document the demyelination of the bilateral pyramidal tracts and parieto-occipital white matter [5, 6]. The *GALC* gene, spanning 60 kb of genomic DNA on chromosome 14q31, encodes the enzyme β-galactocerebrosidase, which is critical for glycosphingolipid catabolism [7]. According to the Human Gene Mutation Database (HGMD), more than 130 mutations have been catalogued. At least 128 of them were viewed as being pathogenic of Krabbe disease [8].

Here we report the *GALC* mutations, a known and a novel one, in Chinese siblings with late-onset Krabbe disease.

## Case presentation

### Patient 1

A 25-year-old male, from China, born to unrelated parents was presented to the First People’s Hospital of Huizhou city, China. The clinical manifestations were spastic gait disturbance and vision loss (Table 1). He was suffering from mild gait difficulties by the age of 5 years; the ambulation was unstable, and he could fall easily. The vision loss was reported at the age of 8 years, while the cognitive development was normal. He was born at full term by uncomplicated delivery. The neurological examination of the patient revealed ocular motility disorders, horizontal nystagmus, absence of the left pupillary light reflex, pes cavus, spastic paraparesis on lower limbs, exaggerated bilateral patellar tendon reflexes, ankle clonus, and positive Babinski sign, while no detectable defect was found in the finger-to-nose test, sensory function. The laboratory biochemical studies of full blood count, liver function, plasma electrolytes, thyroid function, vitamin B-12 and folate, sex hormone, autoantibody profile and syphilis serology exhibited typical levels. Cerebrospinal fluid tests revealed increased protein (1186 mg/L); the normal value was 140–450 mg/L. The GALC enzymatic activity [9] detected by Bio-Tek FLx 800 fluorescent analyzer in leukocytes was decreased (3.9 nmol/mg/17 h); the normal value was 18–75 nmol/mg/17 h protein.

**Table 1:** Timeline of patient 1

1997	mild gait difficulties, unstable ambulation
2000	vision loss
2017	progressive spastic gait disturbance and vision loss

**Fig. 1:**
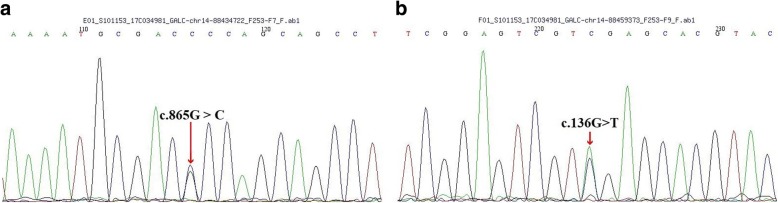
Molecular genetic analysis of the *GALC* gene showed the two mutations, c.865G > C inherited from the patients’ mother (**a**) and c.136G > T inherited from their father (**b**)

**Fig. 2:**
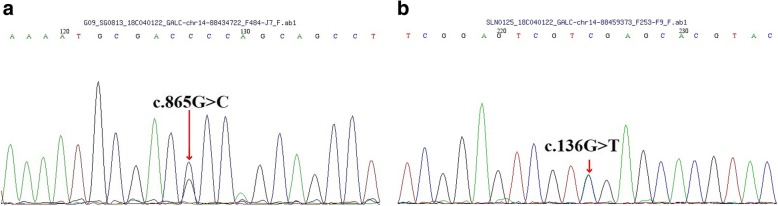
The sister was heterozygous for the *GALC* mutation c.865G > C (**a**) and heterozygous for c.136G > T (**b**)

The described findings gave reason to perform molecular analysis of the *GALC* gene. The direct sequencing of the *GALC* gene (Reference mRNA sequence: NM_000153) in this patient identified a novel missense mutation (c.865G > C: p. G289R) in exon 8 along with a known missense mutation [10] (c.136G > T: p. D46Y) in exon 1 (Figs. 1 and 2). The former mutation was heterozygous in the mother, while the latter was heterozygous in the father.

**Fig. 3:**
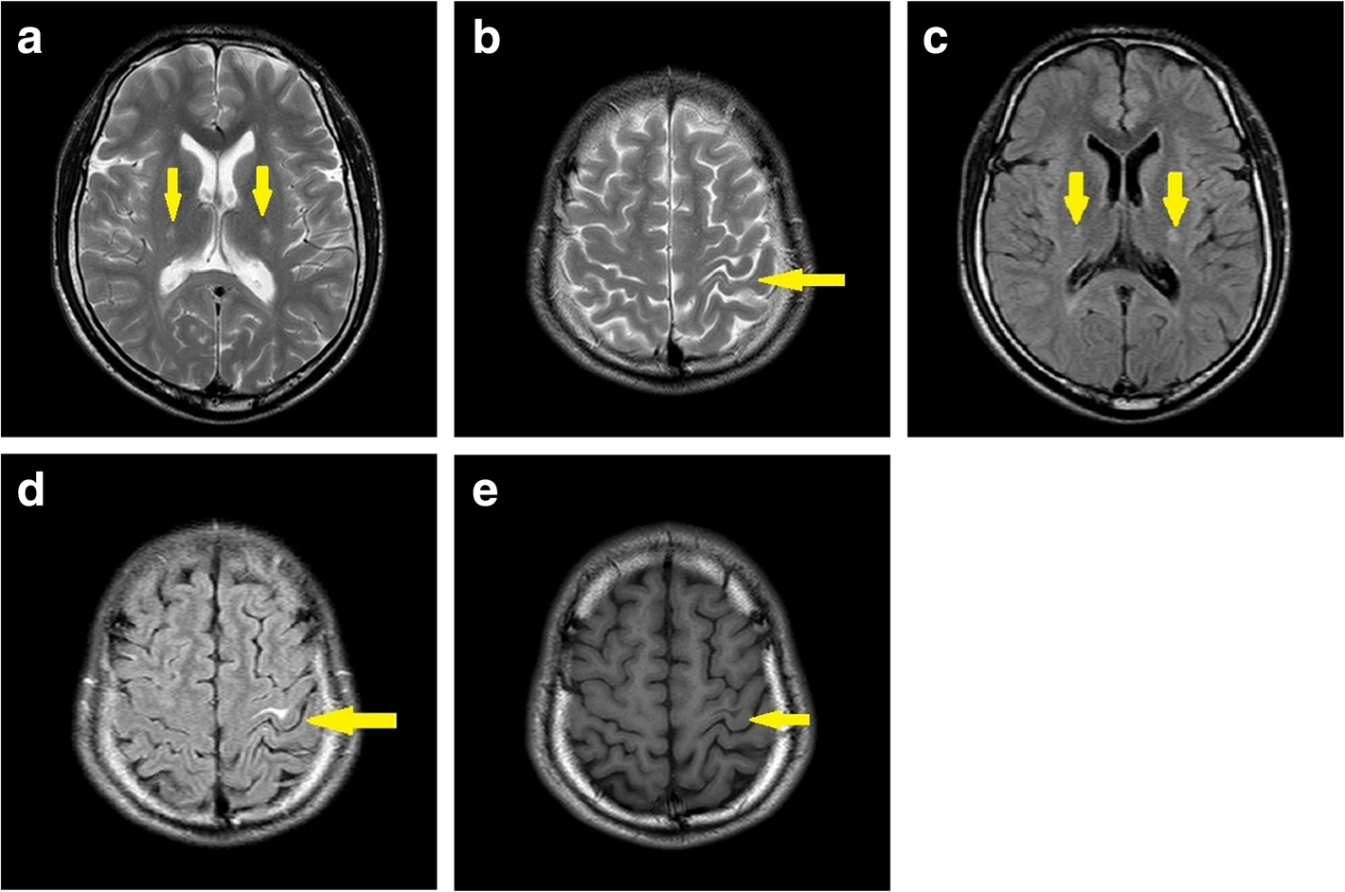
Cerebral MR scans of the patient 1. The axial T2w and FLAIR image showed bilateral corticospinal tracts (**a** and **c**) and the left central gyrus cortex (**b** and **d**) signal hyperintensities. Cerebral T1w axial images (**e**) revealed a decreased signal in the left central gyrus cortex

**Fig. 4:**
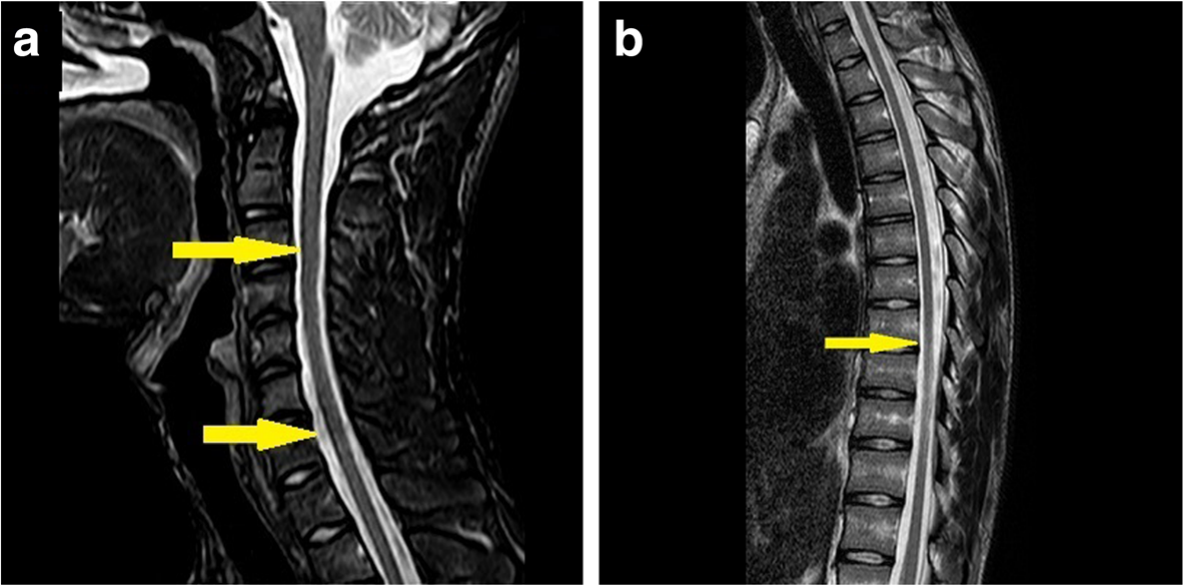
Spinal T2w axial images (**a** and **b**) showed mild atrophy of spinal cord

Brain MRI revealed a high-intensity signal in the left central gyrus cortex by fluid-attenuated inversion recovery (FLAIR) as well as T2-weighted images, while a decreased signal in the T1-weighted images and high-intensity lesions in the bilateral corticospinal tracts were detected (Fig. 3). Cervical spine and thoracic spine MRI showed mild atrophy of the spinal cord (Fig. 4). Moreover, electromyography indicated peripheral nerve demyelination, and both visual evoked potentials (VEP) and brainstem auditory evoked potential (BAEP) were normal.

### Patient 2

**Fig. 5:**
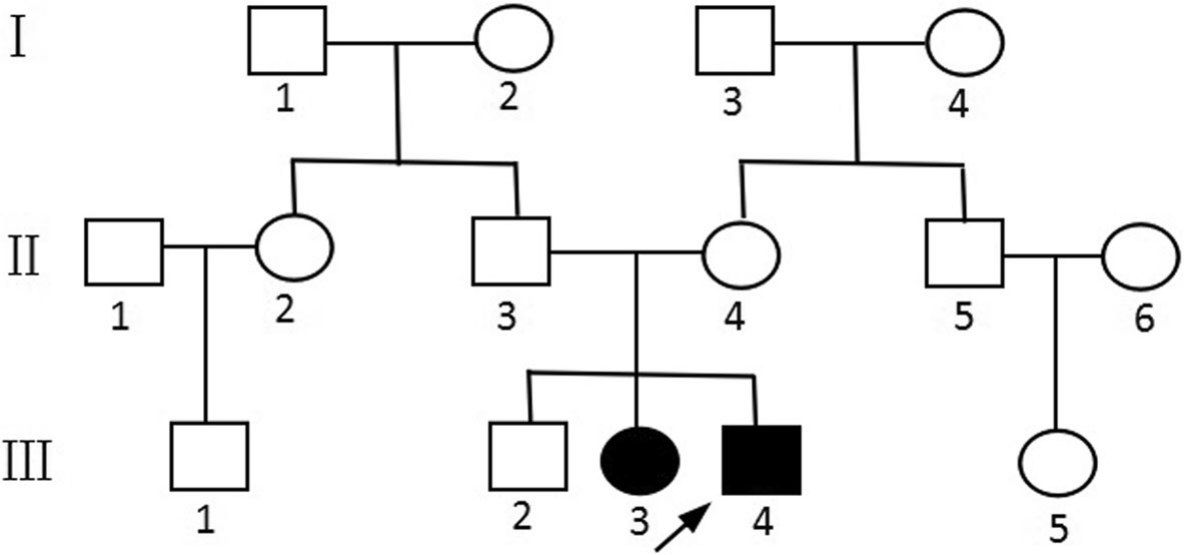
Pedigree of the family

In the family history, his elder sister’s development was normal until the age of 4 years when spastic gait disturbance and dysarthria were noticed. She suffered from mental and motor regression, and hence, faced difficulties in schooling. She became wheelchair-bound and demented at the age of 22 years. She could not understand what was said to her but responded with a smirk. Other family members, including her parents and elder brother, were unaffected at the time of this analysis (Fig. 5). The GALC enzymatic activity revealed 4.4 nmol/mg/17 h. The *GALC* genotype was also studied in Patient 2. She carried two heterozygous *GALC* mutations (p.G289R and p.D46Y), the same as the Patient 1 (Fig. 2).

The mutations were analyzed to assess their pathogenicity. The SIFT scores of the mutations were 0.003 (p.G289R) and 0.013 (p.D46Y), respectively, and the Polyphen2 scores were 0.905 (p.G289R) and 1 (p.D46Y), respectively.

Thus, Krabbe disease was diagnosed in both patients. Since effective therapy is limited, Patient 1 was treated with neuro-nutrition drugs, such as 30 mg per day vitamin B-1 and 1.5 mg per day vitamin B-12 for about 1 month. But his condition was not relieved.

## Discussion and conclusions

Several Chinese cases were reported, and more were scattered cases. A study investigated the clinical symptoms of 22 unrelated Chinese patients diagnosed with Krabbe disease. They found the late-onset form of Krabbe disease was more prevalent kind in patients [11].

We described two individuals from a Chinese family affected with spastic paraparesis. Herein, we made a comparison between the two patients’ clinical presentation and with other published Chinese cases [10–21] (Table 2). Motor regression, spasticity, hearing and vision impairment, irritability and excessive crying presented in the early-infantile patients. Mental and motor regression, vision impairment were the main symptoms of the late-infantile patients. The juvenile patients had walking impairment and mental regression as main symptoms. The adult patients were heterogeneous with various symptoms included spastic gait disturbance, hemiplegia, vision impairment, aphasia and mental regression. Comparison to the Patients 1, the Patient 2 had severer symptoms. By the age of 22, she had become wheelchair-bound and demented.

**Table 2:** Clinical summaries of 41 Chinese Krabbe disease patients

Patient No.	Sex	Age of Onset	Main symtoms	Reference
1	F	2 W	cry less, eat less, move less, poor response to environment	[11]
2	M	2 M	lose the ability to hold up his head, mental and motor regression	[16]
3	M	3 M	lose the ability to hold up his head, hearing impairment, irritability and excessive crying	[20]
4	F	4 M	lose the ability to hold up her head, irritability and excessive crying	[12]
5	M	4 M	lose the ability to hold up his head, irritability and excessive crying	[18]
6	F	4 M	Psychomotor regression,dyspepsia, feeding difficulties, irritability,hearing and vision impairment	[11]
7	F	4 M	irritability, feeding difficulties, vision impairment, convulsion opisthotonus	[11]
8	M	6 M	Paroxysmal rigidity of the extremities, mental regression	[11]
9	F	7 M	psychomotor regression	[11]
10	M	8 M	developmental delay, hypertonia of the extremities, vision impairment	[11]
11	F	10 M	motor regression, language development delay, hearing and vision impairment	[11]
12	F	1Y	motor regression, rigidity	[12]
13	F	1Y	language development delay, muscle weakness	[11]
14	M	1Y2 M	psychomotor regression, language development delay	[11]
15	M	15 M	muscle weakness, walking impairment	[11]
16	F	2Y	mental and motor regression, vision impairment	[12]
17	M	2Y	mental and motor regression, vision impairment	[15]
18	M	2Y	psychomotor regression, rapid vision loss	[11]
19	F	2Y5M	seizure, psychomotor regression	[11]
20	M	2Y8 M	mental and motor regression	[19]
21	F	3Y2 M	psychomotor regression, vision impairment, dyspepsia, feeding difficulties	[11]
22	M	3Y5M	motor regression, seizure	[13]
23	M	3Y11M	walking impairment	[11]
24	F	4Y	mental regression, motor regression, spastic gait disturbance and dysarthria	Patient 2
25	M	5Y	spastic gait disturbance and vision impairment	Patient 1
26	M	5Y	weakness of both lower limbs	[15]
27	M	5Y	muscle weakness, walking impairment	[11]
28	M	8Y10 M	walking and vision impairment	[11]
29	M	12Y	spastic gait disturbance, weakness of both lower limbs	[21]
30	M	20Y	weakness of left lower limb	[17]
31	F	20Y	psychomotor regression, aphasia	[11]
32	F	29Y	weakness of right lower limb	[20]
33	F	30Y	spastic gait disturbance,weakness of both lower limbs	[21]
34	F	37Y	weakness of the left upper limb, walking impairment	[10]
35	F	38Y	weakness of both lower limbs, rigidity	[14]
36	M	45Y	numbness and weakness of both lower limbs, rigidity	[14]
37	M	unknown	motor regression	[11]
38	F	unknown	psychomotor regression	[11]
39	M	unknown	mental regression, walking impairment, hearing and vision impairment	[11]
40	M	unknown	left limb movement disorder	[11]
41	M	unknown	walking impairment	[11]

In the Patient 1, electromyography indicated peripheral nerve demyelination, while brain MRI showed an increased signal intensity in the white matter encompassing the bilateral corticospinal tracts. The GALC enzymatic activity in leukocytes was 3.9 nmol/mg/17 h. In the Patient 2, the GALC enzymatic activity revealed 4.4 nmol/mg/17 h. These low activities proposed the diagnosis of Krabbe disease in both patients.

Furthermore, *GALC* gene mutations were found in the patients. The first novel mutation was a single nucleotide substitution (c.865G > C) in exon 8 of the *GALC* gene, resulting in glycine substitution to arginine at position 289 (p. G289R). This mutation was heterozygous in the proband’s mother. The second mutation (c.136G > T) found in our patient was also a single nucleotide substitution in exon 1, which caused a substitution of aspartic acid to tyrosine at position 46 (p. D46Y). The two patients inherited this mutation from their father, who was heterozygous for the mutation. Interestingly, this mutation was reported in another patient with adult-onset, although no vision loss was reported [10, 22].

The phenotype and genotype for the Krabbe disease show considerable variation worldwide, thus rendering difficulty in accurate diagnosis [23]. The differential diagnosis includes hereditary spastic paraplegia, Charcot-Marie-Tooth disease, and Kennedy disease. Brain MRI and GALC activity assay are essential for patients manifesting chronic progressive corticospinal tract impaired. However, the relationship between the deficiencies in the lysosomal enzyme activities and the degree of clinical severity appears obscure [21, 24]. Both the two mutations are reported in the gnomad dataset (http://gnomad.broadinstitute.org). As there is no prior case report of Krabbe patients carrying the mutation (c.865G > C: p.G289R), the allele frequency information for p.G289S from gnomad is 4.067e-6, which suggests the mutation might be probably damaging. Furthermore, based on SIFT and Polyphen2, we suggest that the two mutations (c.865G > C: p.G289R and c.136G > T: p.D46Y) were “likely pathogenic.”

The present study describes two *GALC* mutations shared by two Chinese siblings with juvenile-onset of Krabbe disease and decreased GALC enzyme activity. These mutations might contribute towards increasing the public awareness about Krabbe disease and enriching the pathogenic database of *GALC*.

## Data Availability

The authors declare that all the data are contained within the manuscript.

## Abbreviations

BAEP: Brainstem auditory evoked potential
FLAIR: Fluid-attenuated inversion recovery
GALC: Galactocerebrosidase
HGMD: Human Gene Mutation Database
MRI: Magnetic resonance imaging
VEP: Visual evoked potentials
